# Death of Cuvier

**Published:** 1832-06

**Authors:** 


### OBITUARY.

*Death of* Cuvier.

Science and literature have lost one of their brightest ornaments: the well-known naturalist Baron Cuvier died at Paris on the 16th instant. The day before his demise, he was attacked with apoplexy and paralysis. He was at the time actively engaged in the completion of several important works, particularly his splendid work on Fishes, and his Comparative Anatomy.

Cuvier was the son of a Protestant clergyman, and was born in 1769. He so freely expended in scientific pursuits the emoluments he derived from his distinguished labours, that his family are left almost unprovided for. The French government, we are happy to learn, have testified their high respect for his memory by giving to his widow a handsome annuity. The distinguished talent of Baron Cuvier as a naturalist was known and duly appreciated throughout the world; and to his wonderful attainments as a philosopher he added that of a most impressive and eloquent writer. We know no more pleasing work in the French language than that by Baron Cuvier, entitled "Eloges Historiques des Membres de l'Académie Royale des Sciences."

It is often the case that men, who distinguish themselves by extraordinary talents, disgrace their studies by their vices; but Cuvier was as much esteemed for his moral worth as he was for his profound abilities. His works secured him the veneration of those who were strangers to his estimable qualities as a man, while those who were personally acquainted with him felt for him the sincerest affection.

A short time since, Pickersgill, when in Paris, painted the portrait of Cuvier: it is still unfinished in the unimportant details, but the face is fortunately complete. We saw this portrait, which, through the kindness of the artist, was exhibited last Friday week at the Royal Institution. It is an admirable painting, and a most faithful likeness. We hope it will be purchased for some of our public institutions; and, in our opinion, no fitter place could be found for it than the museum of the College of Surgeons.

We trust that, upon some future occasion, we shall be able to present our readers a more satisfactory account of the life and labours of the great Cuvier. Some kindred genius will doubtless give to the world the memoirs of this extraordinary man, whose discoveries have shed such a brilliant lustre upon the age in which he lived.

### MONTHLY LIST OF MEDICAL BOOKS.

